# The effect of esketamine on postoperative delirium in patients undergoing general anesthesia: a systematic review and meta-analysis

**DOI:** 10.3389/fphar.2025.1681531

**Published:** 2025-11-07

**Authors:** Chao-Xu, Rui-Jun Tong, Qi-Hong Shen, Jing Zhao

**Affiliations:** 1 Department of Anesthesiology, Jiashan First People’s Hospital, Jiaxing, China; 2 Department of Anesthesiology, First Hospital of Jiaxing, Jiaxing, China

**Keywords:** esketamine, postoperative delirium, meta-analysis, POD, postoperative nausea and vomiting (PONV)

## Abstract

**Background:**

Postoperative delirium (POD), a prevalent neurological complication, is strongly associated with adverse clinical outcomes. This meta-analysis aimed to evaluate the efficacy of esketamine in preventing POD among patients receiving general anesthesia.

**Methods:**

We systematically searched PubMed, Embase, Cochrane Library, Web of Science, clinical trial registries and major conference proceedings for randomized controlled trials (RCTs) examining esketamine’s impact on POD in general anesthesia patients, from inception through 30 June 2025. Statistical analyses were performed using RevMan 5.4 and Stata 12.0. Dichotomous outcomes were expressed as risk ratios (RR) with 95% confidence intervals (CI), while continuous variables were analyzed via mean differences (MD). Study bias was assessed with the Cochrane ROB 2.0 tool.

**Results:**

Thirteen RCTs involving 1,873 elective surgery patients under general anesthesia were included. Esketamine administration was associated with a lower POD incidence (RR: 0.66; 95% CI: 0.49–0.91; P < 0.05). Subgroup analyses revealed potentially significant reductions in adult populations and cardiac surgery cohorts. The postoperative nausea and vomiting (PONV) rate decreased in the esketamine group. Additionally, esketamine was associated with reduced pain scores at 24 h postoperatively.

**Conclusion:**

Our findings suggest that esketamine may be associated with a lower POD risk following general anesthesia. Further large-scale trials are warranted to validate these preliminary findings.

**Systematic Review Registration:**

https://www.crd.york.ac.uk/PROSPERO/recorddashboard.

## Introduction

Delirium manifests as an acute cognitive disturbance characterized by fluctuating impairments in attention and awareness. Postoperative delirium (POD), typically emerging on postoperative days 2–5 (Jin et al., 2020), is a recognized predictor of delayed neurocognitive recovery (Singh et al., 2024). This complication correlates with elevated mortality risks, increased postoperative morbidity, unplanned ICU admissions, prolonged hospitalization, and higher healthcare costs (Feng et al., 2025; Ron and Deiner, 2024). Risk factors encompass patient-specific variables (e.g., advanced age, preexisting cognitive impairment, polypharmacy, comorbidities), anesthesia/surgical parameters (e.g., extended anesthesia duration), and postoperative events (e.g., infections, respiratory complications) (Shen et al., 2025; Sun F. et al., 2025; Zhou et al., 2025).

Recent pathophysiological models implicate microglia-driven neuroinflammation as a primary mechanism underlying POD (Mu et al., 2022; Alam et al., 2018). Ketamine—an NMDA receptor antagonist with sedative-analgesic properties—exerts neuroprotective effects via mitigation of excitotoxicity and microglial modulation (Ho et al., 2019). Although its non-anesthetic applications represent novel therapeutic avenues, ketamine’s efficacy for POD prophylaxis remains controversial, and a 2023 meta-analysis showed no preventive benefit (Fellous et al., 2023).

As the S (+) enantiomer of ketamine, esketamine demonstrates higher NMDA receptor affinity, enhanced analgesic potency, accelerated metabolic clearance, and improved safety profiles compared to racemic ketamine (Wang et al., 2019). Emerging evidence suggests that intraoperative esketamine may reduce POD incidence in general anesthesia settings (Zhang W. et al., 2024); however, limitations in existing studies include inconsistent delirium assessment methodologies. Notably, recent clinical trials reported no significant POD reduction with esketamine (Zhao et al., 2025; Zhang Y. et al., 2024).

So far, no meta-analysis has been published specifically regarding esketamine for preventing POD. Previous studies mainly focused on postoperative depression (Sun X. et al., 2025; Wen et al., 2025). The present study strictly included only randomized controlled trials (RCTs) that applied standardized, well-defined criteria for POD diagnosis. This meta-analysis aimed to evaluate the preventive efficacy of perioperative esketamine versus placebo or non-intervention for POD.

## Methods

### Study design and registration

This meta-analysis was pre-registered in PROSPERO (CRD420251060356) and conducted in strict adherence to the PRISMA 2020 guidelines, ensuring a systematic and transparent approach to the review process. The protocol can be accessed at: https://www.crd.york.ac.uk/PROSPERO/recorddashboard.

### Information sources and search strategy

A thorough and systematic search was performed across four major electronic databases (PubMed, Embase, Cochrane Library, and Web of Science), which encompassed records from the inception of each database up to 30 June 2025, with no language restrictions applied. In PubMed, the search strategy included the following terms: {[“delirium”(MeSH Terms) OR “delirium”(All Fields)]} AND (“esketamine”[MeSH Terms] OR “esketamine”[All Fields]). Only randomized controlled trials (RCTs) involving adult participants were considered. Furthermore, to minimize publication bias, our search strategy also included clinical trial registries (ClinicalTrials.gov, WHO ICTRP) and major conference proceedings (e.g., the American Society of Anesthesiologists Annual Meeting) for ongoing or unpublished studies. Additionally, reference lists of included studies were manually reviewed to identify potentially relevant articles, and corresponding authors were contacted to obtain unpublished data where necessary.

### Study selection criteria

The inclusion criteria were defined using the PICOS framework as follows: Population (P): Adults aged 18 and older undergoing elective procedures under general anesthesia; Intervention (I): Esketamine; Comparator (C): Placebo or no intervention; Outcome (O): Primary outcome - incidence of POD; secondary outcomes - postoperative pain scores, and incidence of postoperative nausea and vomiting (PONV); Study design (S): Parallel-group RCTs. Exclusion criteria comprised: (1) Non-randomized designs (e.g., case series, editorials, narrative reviews); (2) Conference abstracts lacking peer-reviewed full texts; (3) Ongoing trials without available primary outcome data; (4) Studies lacking a clearly standard definition of POD.

### Data extraction protocol

Two independent researchers (CX and RJT) conducted the study selection process: they first removed duplicate records using EndNote, then performed title and abstract screening to assess relevance. Full-text articles were then reviewed to determine eligibility based on the inclusion criteria. The inter-rater agreement for full-text screening was excellent (Cohen’s kappa = 0.85). Data extracted from each study included: study details (authors, publication year, sample size), patient demographics (age), type of surgery, esketamine dosage and administration timing. Also, we extracted data on the specific POD assessment tool used (e.g., confusion assessment method (CAM), CAM-ICU, 3D-CAM), the frequency of assessments, and the total postoperative observation period for each study. Any disagreements were resolved through discussion or by consulting a third reviewer (JZ).

### Risk of bias and evidence quality assessment

The risk of bias in included studies was assessed using the Cochrane risk-of-bias tool for RCTs (ROB 2.0), which evaluates five areas: (1) bias arising from the randomization process; (2) bias due to deviations from intended interventions; (3) bias due to missing outcome data; (4) bias in measurement of the outcome; and (5) bias in selection of the reported result. Particular attention was paid to the measurement of POD, a subjective outcome, where lack of blinding of outcome assessors was considered a potential source of bias. Each area was classified as low, high, or some concerns based on predefined criteria.

The GRADE framework was used to assess the certainty of the evidence across six domains: study design, risk of bias, imprecision, inconsistency, indirectness, and other considerations. Evidence quality was rated as very low, low, moderate, or high.

### Statistical analysis

Meta-analysis was performed using Review Manager 5.3 and Stata version 12.0. For dichotomous outcomes, pooled effects were calculated as risk ratios (RR) with 95% confidence intervals (CIs). Continuous outcomes were analyzed using standardized mean differences (SMD) or weighted mean differences (MD), with 95% CIs. Two studies reporting postoperative pain scores, Jing et al. (2024) and Zhang C. L. et al. (2024), presented data as median and interquartile range. These values were converted to mean and standard deviation using the validated methods described by Luo et al. (2018), Wan et al. (2014) to permit meta-analysis. Statistical significance was set at α = 0.05. Between-study heterogeneity was quantified using I^2^ statistics, with values over 50% indicating substantial heterogeneity. A random-effects model was used for all analyses due to clinical heterogeneity in surgical protocols and analgesic regimens. Subgroup analyses were performed based on patient age (adults vs. elderly) and type of surgery (non-cardiac vs. cardiac). Publication bias was assessed using funnel plots, and sensitivity analysis was conducted to evaluate the robustness of the primary outcome. All subgroup and sensitivity analyses were considered exploratory and hypothesis-generating. No statistical adjustments for multiple testing were performed, which increases the risk of type I errors. Findings from these analyses should therefore be interpreted with caution. For studies with missing or unclear data regarding our primary or secondary outcomes, we attempted to contact the corresponding authors via email to request the necessary information. If no response was received after two attempts, the study was included only for the outcomes with available and clearly reported data, and this was noted during the analysis.

Trial sequential analysis (TSA) was used to control for the risk of Type I error from multiple comparisons using TSA software (version 0.9.5.10 beta). Further research was deemed unnecessary when the cumulative z-curve crossed the TSA monitoring boundary or reached the required information size (RIS). The risk of Type I error was set at 5% with a power of 80%.

### Ethical considerations

This systematic review and meta-analysis, being a secondary analysis of previously published data, did not require separate ethical approval. However, we confirm that all included primary studies reported obtaining approval from their respective institutional review boards or ethics committees, as well as informed consent from participants.

## Results

### Search results

**FIGURE 1:**
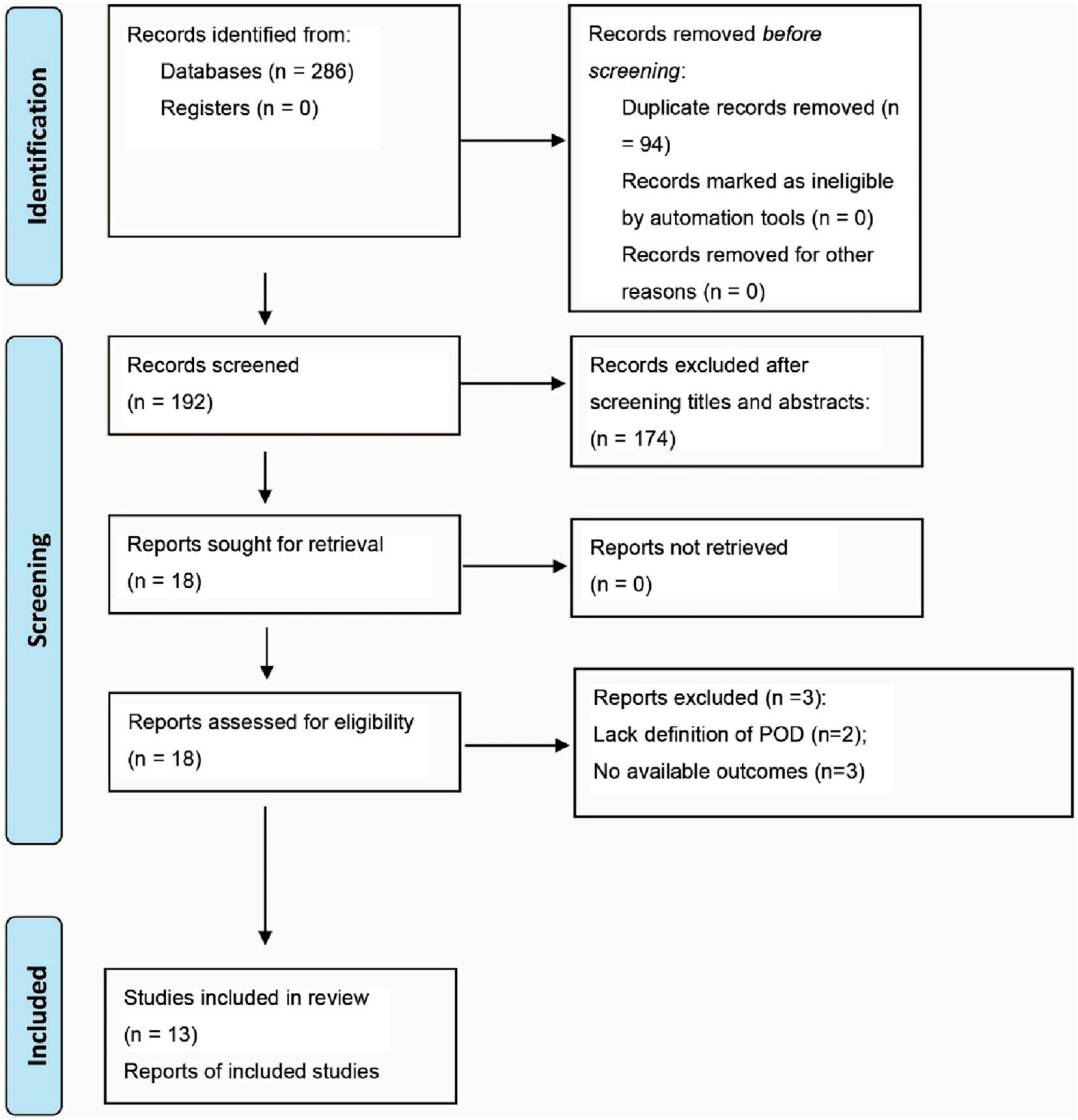
Literature search inclusion process.

A systematic search identified 286 potential records as of 30 June 2025. After removing 94 duplicate records, 174 were excluded based on title and abstract screening. The remaining 18 studies underwent full-text review, resulting in exclusions based on the PICOS criteria: three studies lacked adequate outcome reporting (Bornemann-Cimenti et al., 2016; Lu et al., 2024; Ye et al., 2024); and two failed to define POD (n = 2) (Chen et al., 2022; Han et al., 2023). Ultimately, 13 RCTs were included in the synthesis (Zhao et al., 2025; Zhang Y. et al., 2024; Jing et al., 2024; Zhang C. L. et al., 2024; Huang et al., 2024; Ju et al., 2025; Li et al., 2022; Liu et al., 2024; Liu et al., 2023; Luo et al., 2024; Ma et al., 2024; Ma J. et al., 2023; Xiong et al., 2024), with the selection process detailed in the PRISMA 2020 flowchart (Figure 1).

### Study characteristics

The 13 RCTs included 1,873 participants, with 965 in the esketamine group and 908 in the control group. These studies were published between 2022 and 2025, with sample sizes ranging from 39 to 209. All studies were conducted in China. Detailed characteristics of the included RCTs are presented in Table 1.

**TABLE 1:** The details of included studies.

Study	Age	Sample size	ASA scale	Type of surgery	Esketamine group	Control group	Diagnostic tools of POD	Anesthesia induction method
Huang2024	≥60	209	I–III	Orthopedic, urologic and major abdominal surgeries	Dosage: 0.5 mg/kg Timing: after anesthesia induction Route: intravenous	A volume of saline equivalent to 0.05 mL/kg was injected after the induction	CAM scale twice daily for 7 days postoperatively	Midazolam 0.02–0.05 mg/kg, propofol 1.5–2 mg/kg, sufentanil 0.4 μg/kg, and rocuronium 0.6 mg/kg
Jing2024	≥60	87	I–III	Laparoscopic gastrointestinal tumor surgery	Dosage: 0.5 mg/kg Timing: after anesthesia induction Route: intravenous	An equivalent volume of normal saline	CAM scale twice daily for 3 days postoperatively	Midazolam 0.04 mg/kg, propofol 1.5 mg/kg, sufentanil 0.5 μg/kg and rocuronium 0.9 mg/kg
Ju2025	≥18	134	Ⅲ-Ⅳ	Off-pump coronary artery bypass grafting	Dosage: 0.25 mg/kg/h Timing: during anesthesia maintenance Route: intravenous	An equal volume of normal saline	CAM-ICU or 3D-CAM scale twice daily for 7 days postoperatively	Midazolam 0.05 mg/kg, etomidate 0.2 mg/kg, sufentanil 1–3 μg/kg, and cisatracurium 0.2–0.3 mg/kg
Li2022	65–85	80	Ⅱ-III	Unilateral total knee arthroplasty	Dosage: 0.2 mg/kg Timing: after anesthesia induction Route: intravenous	An equal volume of normal saline	CAM scale at 24 h and 72 h after surgery	Sufentanil 0.4–0.5 μg/kg, etomidate 0.2–0.3 mg/kg, rocuronium 0.6 mg/kg
Liu2024	≥65	60	I–III	Laparoscopic gastrointestinal surgery	Dosage: 1 mg/kg Timing: after surgery Route: intravenous by PCA	PCA with sufentanil 2 ug/kg	CAM at the 1st and 3rd postoperatively, frequency was not reported	Midazolam 0.05 mg/kg, etomidate 0.3 mg/kg, sufentanil 0.4 μg/kg, and rocuronium bromide 0.8 mg/kg
Liu2023	18–60	39	I–II	Laparoscopic gynecological surgery	Dosage: 0.125 mg/kg Timing: after the start of surgery Route: intravenous	An equal volume of normal saline	CAM-ICU. assessment timing and frequency were not reported	Propofol, 1–2 mg/kg, sufentanil 0.2–0.4 μg/kg, and rocuronium 0.6 mg/kg
Luo2024	Adult	129	I–III	Resection of lung or mediastinal tumors	Dosage: 0.2 mg/kg or 0.5 mg/kg Timing: during anesthesia Route: intravenous	An equal volume of normal saline	CAM scale at the 1st and 3rd day after surgery, frequency was not reported	Midazolam 2 mg, etomidate 0.3 mg/kg, sufentanil 0.4–0.6 µg/kg and rocuronium 0.6–0.9 mg/kg or cisatracurium 0.2 mg/kg
Ma2024	≥60	260	Ⅱ-III	Total hip arthroplasty or total knee arthroplasty	Dosage and timing: 0.20 mg/kg for induction and 0.125 mg/kg/h for maintenance Route: intravenous	An equal volume of normal saline	3D-CAM twice daily during the first 3 postoperative days	Etomidate 0.3 mg/kg, alfentanil 40–50 ug/kg, and rocuronium 0.6 mg/kg
Ma2023	≥65	62	Ⅰ-III	Major abdominal surgery	Dosage and timing: 0.25 mg/kg for induction and 0.125 mg/kg/h for maintenance Route: intravenous	An equal volume of normal saline	CAM-ICU 1–3 d after surgery, frequency was not reported	Sufentanil 0.25–0.5 ug/kg, etomidate 0.15–0.3 mg/kg, cisatracurium 0.15–0.2 mg/kg
Xiong2024	≥18	42	Ⅱ-III	Cardiac valve replacement surgery	Dosage: 0.25 mg/kg Timing: before anesthesia induction Route: intravenous	An equal volume of normal saline	CAM or CAM-ICU twice daily for 7 days postoperatively	Midazolam 0.05 mg/kg intravenous, sufentanil 0.5 μg/kg, etomidate 0.3 mg/kg, and rocuronium 0.6 mg/kg
Zhang C2024	65–80	163	Ⅱ-III	Thoracoscopic radical lung cancer surgery	Dosage and timing: 0.25 mg/kg for induction and 0.125 mg/kg/h for maintenance Route: intravenous	An equal volume of normal saline	3D-CAM scale within 7 days after surgery, frequency was not reported	Midazolam 0.05–0.1 mg/kg, etomidate 0.2–0.3 mg/kg, sufentanil 0.3–0.5 μg/kg and rocuronium 1–1.5 mg/kg
Zhang Y2024	65–85	426	Ⅱ-III	Elective noncardiac surgery	Dosage: 0.2 mg/kg Timing: before anesthesia induction Route: intravenous	An equal volume of normal saline	3D-CAM in the morning at 1, 3, and 7 days postoperatively	Propofol, sufentanil and cisatracurium were given as appropriate for induction
Zhao2025	65–80	60	Ⅱ-III	Total hip arthroplasty or total knee arthroplasty	Dosage: 0.72 mg/kg Timing: after surgery Route: intravenous by PCA	PCA with sufentanil 2 ug/kg	3D-CAM at 3rd day after surgery, frequency was not reported	Etomidate 0.2 mg/kg, midazolam 0.05 mg/kg, sufentanil 0.5 μg/kg, and rocuronium 0.3 mg/kg

Abbreviation: ASA, american society of anesthesiologists; POD, postoperative delirium; CAM., confusion assessment method; PCA, patient controlled analgesia.

### Risk of bias

**FIGURE 2:**
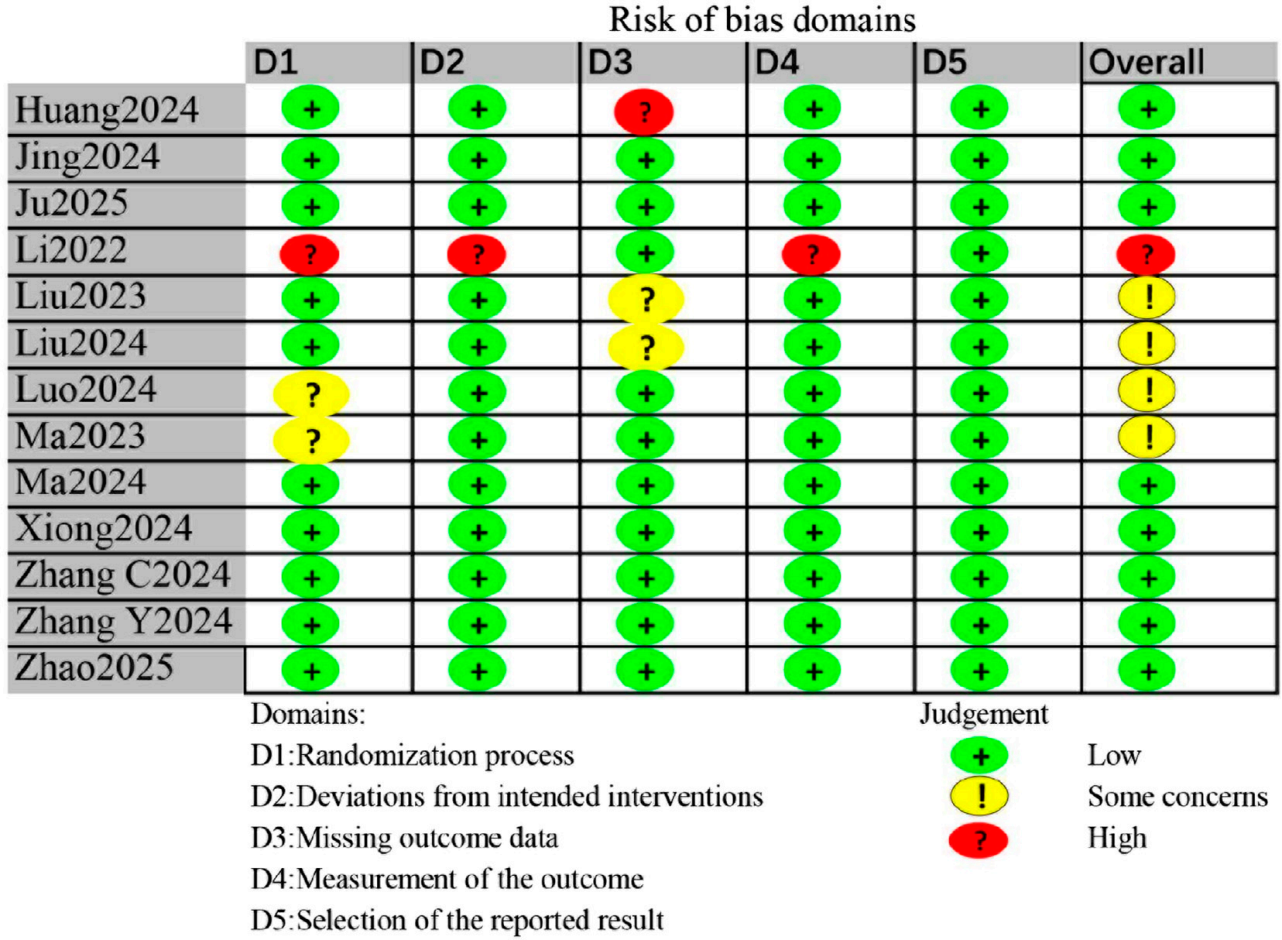
Risk of bias assessment for all included studies.

Risk of bias assessment for individual studies is shown in Figure 2. Three studies were judged as having some concerns or high risk due to issues in the randomization process (Li et al., 2022; Luo et al., 2024; Ma et al., 2024). One study had high risk in the domain of deviations from intended interventions (Li et al., 2022). Among the included studies, the primary concerns regarding risk of bias arose in the domain of measurement of the outcome. Since POD assessment using CAM or CAM-ICU can be subjective, one study that did not explicitly report blinding of the outcome assessor was rated as having high risk in this domain (Li et al., 2022). Of the included trials, eight were classified as low risk of bias, four raised some concerns, and one was considered high risk.

### Primary outcome

**FIGURE 3:**
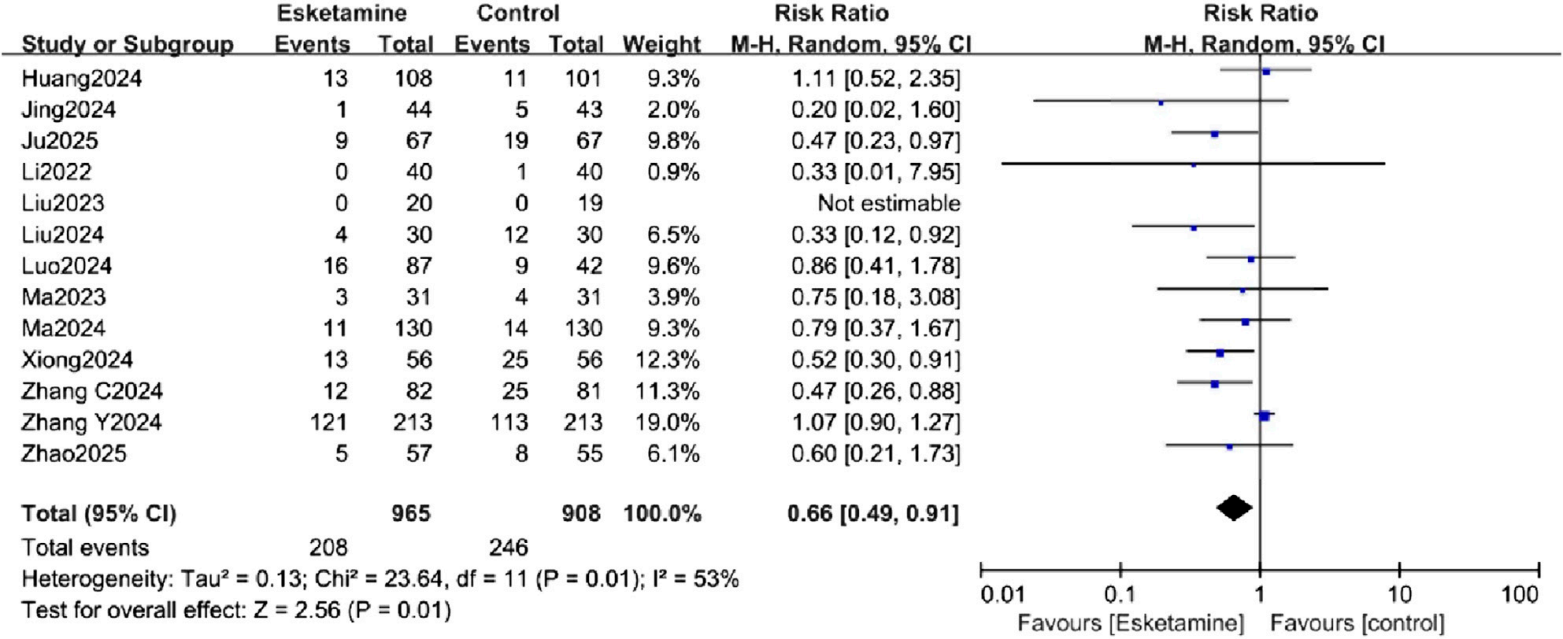
Forest plot comparing the incidence of postoperative delirium (POD) between the esketamine and control groups.

All studies assessed the incidence of POD, measured using the Confusion Assessment Method (CAM) or CAM-ICU. The forest plot demonstrated a significantly lower incidence of POD in the esketamine group (RR = 0.66, 95% CI [0.49, 0.91], P < 0.05, I^2^ = 53%, Figure 3), with high heterogeneity. The absolute risk reduction (ARR) was 5.53%, corresponding to a number needed to treat (NNT) of 18 to prevent one case of POD. Although the pooled analysis suggests a beneficial effect of esketamine, it is crucial to note that the certainty of this evidence was rated as low according to the GRADE framework (Table 2). This downgrading was primarily due to inconsistency (substantial heterogeneity, I^2^ = 53%) and publication bias (as suggested by the funnel plot and Egger’s test).

**TABLE 2:** Summary for GRADE assessment.

Outcome	Included studies (n)	Patients (n)	Quality of evidence	Reasons
Incidence of POD	13	1873	⨁⨁○○ LOW	“Inconsistency” and “Other considerations” were downgraded to “serious”
Incidence of PONV	5	551	⨁⨁⨁○ MODERATE	“Other considerations” was downgraded to “serious”
Postoperative 24-h pain score	4	521	⨁⨁⨁○ MODERATE	“Other considerations” was downgraded to “serious”

Abbreviation: POD, postoperative delirium; PONV, postoperative nausea and vomiting.

### Secondary outcomes

#### Incidence of PONV

**FIGURE 4:**
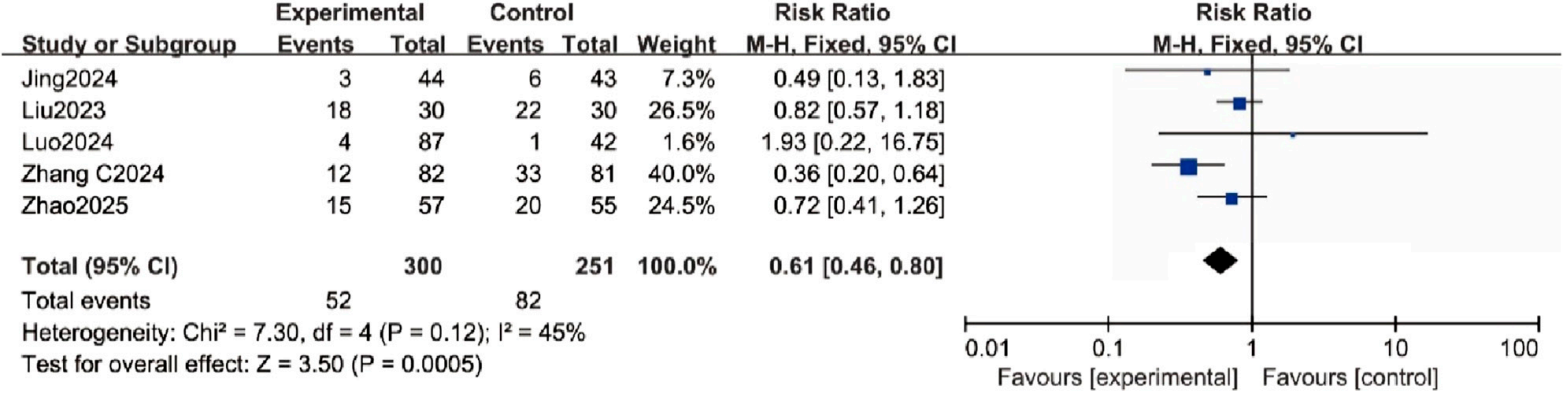
Forest plot comparing the incidence of postoperative nausea and vomiting (PONV) between the esketamine and control groups.

Five trials assessed the incidence of PONV, and the results showed a significantly lower incidence in the esketamine group (RR = 0.61, 95% CI [0.46, 0.80], P < 0.05, I^2^ = 45%, Figure 4), with low heterogeneity.

#### Postoperative 24-hour pain score

**FIGURE 5:**
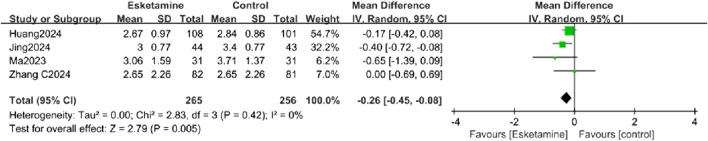
Forest plot comparing the postoperative 24-h pain score between the esketamine and control groups.

Four studies reported postoperative pain scores within 24 h. The forest plot indicated a significantly lower pain score in the esketamine group (MD = −0.26, 95% CI [-0.45, −0.08], P < 0.05, I^2^ = 0%, Figure 5), with low heterogeneity.

#### Subgroup analysis

**FIGURE 6:**
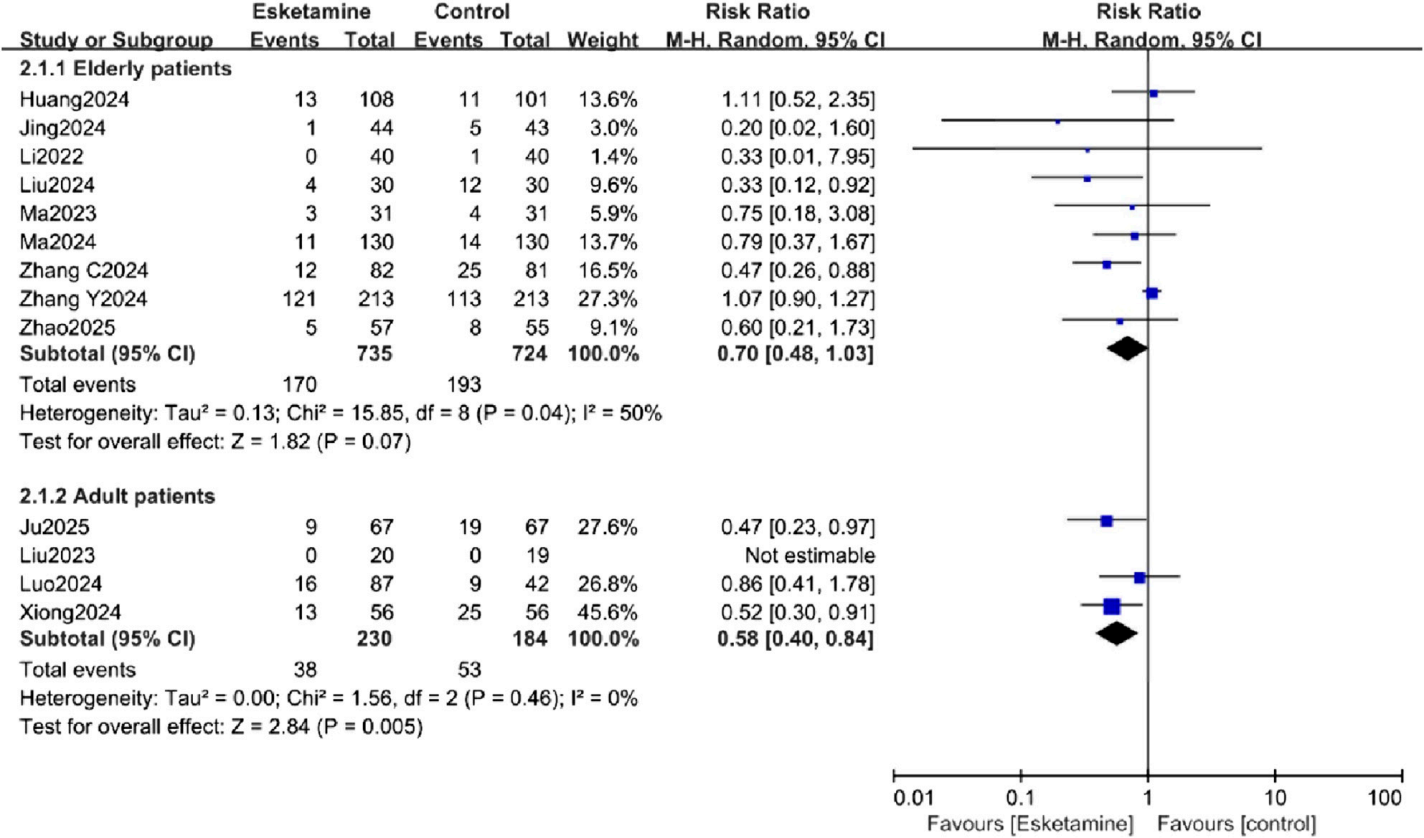
Subgroup analysis of postoperative delirium (POD) incidence by patient age, comparing the esketamine and control groups.

**FIGURE 7:**
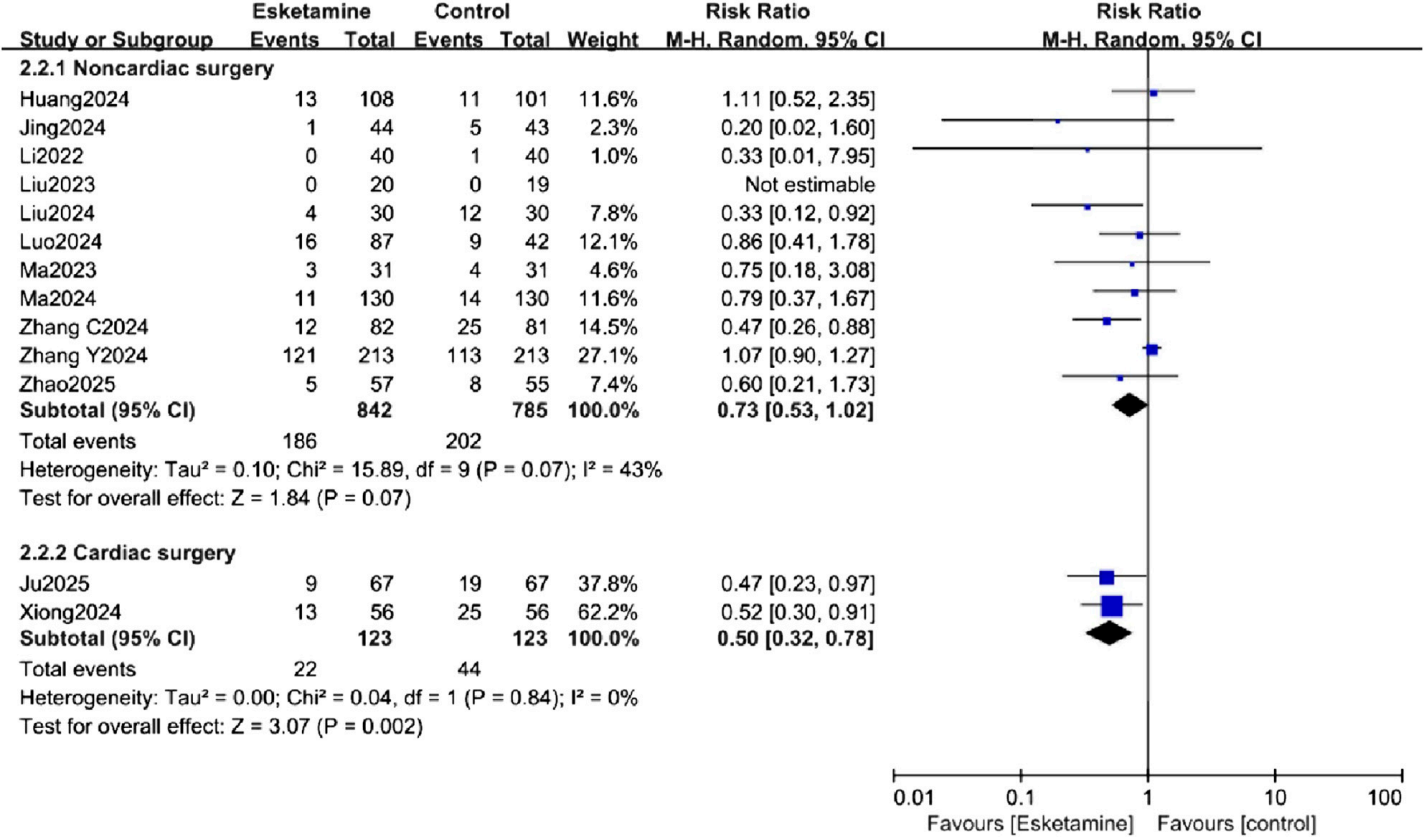
Subgroup analysis of postoperative delirium (POD) incidence by type of surgery, comparing the esketamine and control groups.

Subgroup analyses were conducted to explore sources of heterogeneity for the primary outcome, focusing on patient age (adults vs. elderly) and surgery type (non-cardiac vs. cardiac). The age-related analysis did not significantly reduce the heterogeneity. In the exploratory subgroup analysis of age, a reduction in POD incidence was observed in the adult subgroup, while no difference was observed in the elderly subgroup (Figure 6). The surgery type analysis also did not substantially reduce heterogeneity. Esketamine was effective in reducing POD in the cardiac surgery subgroup, but no significant difference was found in the non-cardiac surgery subgroup (Figure 7).

#### Publication bias and sensitivity analysis

**FIGURE 8:**
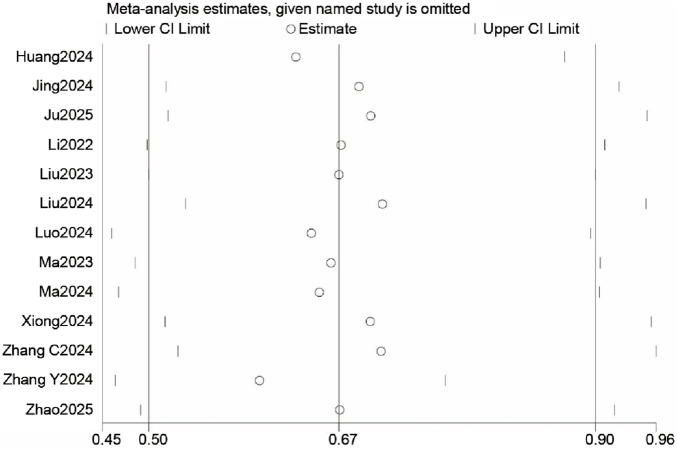
Sensitivity analysis for postoperative delirium (POD) incidence.

The funnel plot (Supplementary Figure S1) showed asymmetry, and the Egger test (P < 0.05) indicated potential publication bias. Sensitivity analysis revealed that the meta-analysis results remained robust, with no significant changes when the number of studies varied, suggesting the stability of the primary outcome (Figure 8).

#### TSA result

**FIGURE 9:**
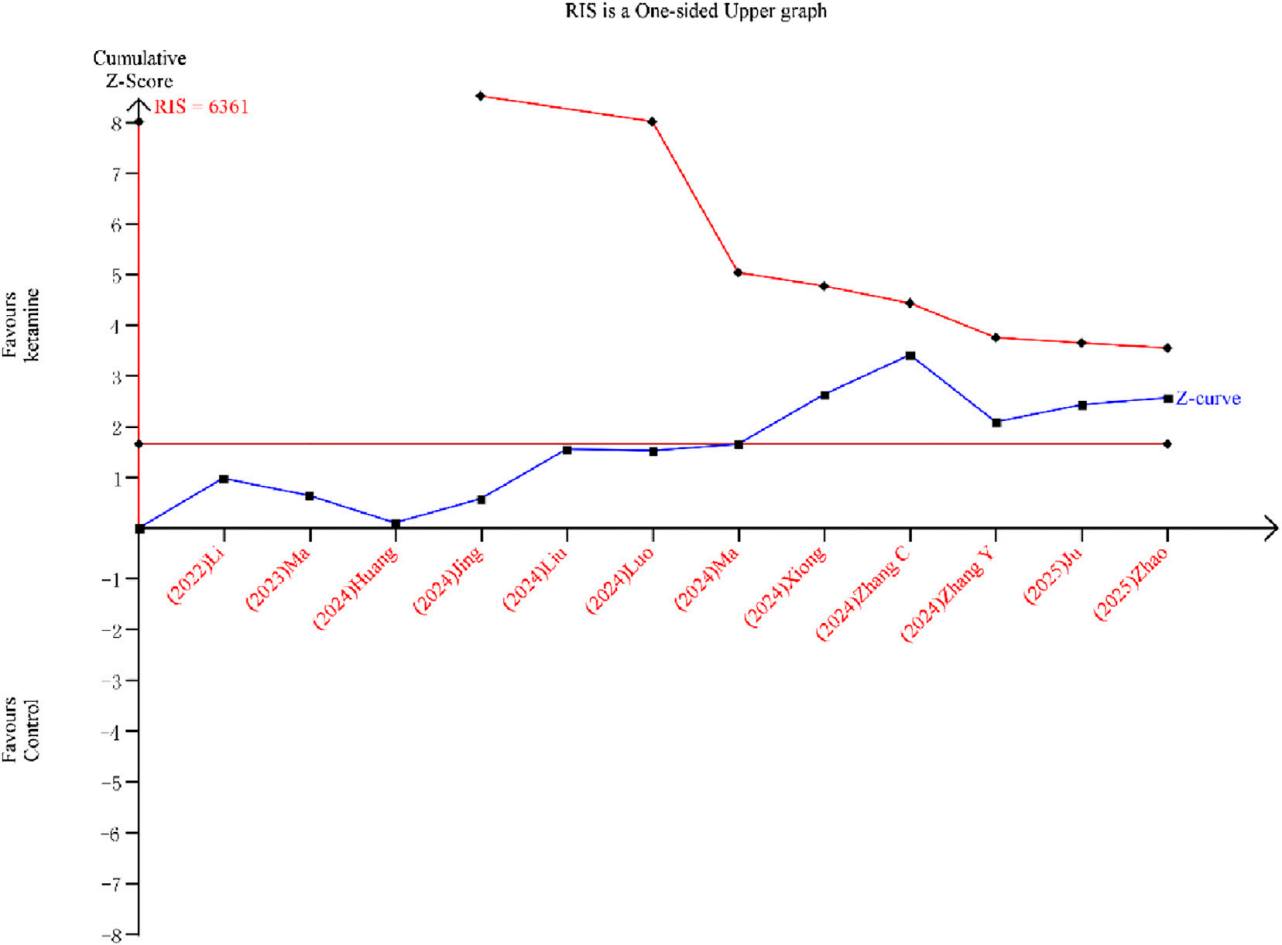
Trial sequential analysis for postoperative delirium (POD) incidence.

Although the cumulative z-curve crossed the traditional boundary, it did not reach the required information size (RIS) line or the TSA monitoring boundary, indicating that the sample size is insufficient for firm conclusions (Figure 9).

#### GRADE result

Our meta-analysis assessed the quality of evidence regarding the impact of esketamine on POD, pain, and PONV using the GRADE approach. The evidence for POD was rated as low, while the evidence for PONV and postoperative 24-h pain scores was rated as moderate. A summary of the GRADE evaluation is presented in Table 2.

## Discussion

This meta-analysis indicated that esketamine reduced the incidence of POD in patients undergoing surgery. Subgroup analyses based on patient age and type of surgery revealed that esketamine significantly decreased POD in adult patients and those undergoing cardiac surgery. Furthermore, esketamine was effective in reducing postoperative pain and PONV incidence. The quality of evidence ranged from low to moderate.

POD is a common complication following surgical anesthesia and has a standardized definition established in 2018 (Evered et al., 2018). Despite extensive research, the exact mechanisms behind POD remain unclear. It is believed that factors such as neuroinflammation, neuroendocrine dysregulation, oxidative stress, blood-brain barrier disruption, synaptic and mitochondrial dysfunction, as well as preoperative and postoperative complications (e.g., Alzheimer’s disease, sleep disorders, pain, and opioid use) contribute to the development of POD (Jin et al., 2020; Guo et al., 2025).

Esketamine, a non-competitive NMDA receptor antagonist, has shown promise in reducing perioperative neurocognitive disorders in recent clinical studies (Lu et al., 2024; Han et al., 2023). However, its effect on POD remains debated. A previous meta-analysis of eight studies suggested that esketamine may reduce POD incidence, but the small sample size and inconsistent POD definitions in the included studies limited the reliability of the findings (Bornemann-Cimenti et al., 2016; Yuan et al., 2022). More recent high-quality clinical trials, however, have found no significant effect of esketamine on POD incidence (Zhao et al., 2025; Zhang Y. et al., 2024). Our meta-analysis aimed to comprehensively evaluate esketamine’s impact on POD, including only studies with clearly defined standard criteria for POD. The potential beneficial effect of esketamine on POD may be mechanistically explained by its dual pharmacological profile. Firstly, as a potent NMDA receptor antagonist, it mitigates glutamate-mediated excitotoxicity and modulates microglia-driven neuroinflammation, both of which are core pathophysiological pathways in the development of delirium (Mu et al., 2022; Alam et al., 2018). Secondly, its established analgesic efficacy, as corroborated by our finding of reduced postoperative pain scores, may indirectly contribute to delirium prevention. Inadequately controlled acute postoperative pain is a well-established risk factor for POD, potentially mediated by stress response activation, sleep disruption, and increased opioid consumption (Ding et al., 2021; Ma J. H. et al., 2023).

In our analysis, a random-effects model revealed that esketamine significantly reduces POD, though with high heterogeneity. Subgroup analyses explored potential sources of this heterogeneity. Unfortunately, heterogeneity did not decrease in these analyses. Notably, esketamine significantly reduced POD in the adult subgroup, but not in the elderly subgroup. It also decreased POD in the cardiac surgery subgroup but showed no effect in the non-cardiac surgery subgroup. The subgroup analyses suggesting greater benefit in adults and cardiac surgery patients are exploratory findings based on a small number of trials. They should be viewed as generating hypotheses for future research rather than as conclusive evidence of differential effects.

Additionally, we found that esketamine aids in postoperative analgesia, supporting previous findings. Hu et al. showed that subanesthetic esketamine improved recovery outcomes in patients undergoing thoracoscopic lung resection (Hu et al., 2025). A recent meta-analysis also demonstrated that intravenous esketamine, as an adjunct to general anesthesia, effectively reduced pain and opioid requirements in the early postoperative period (Wang et al., 2021). Although the pain relief effect is temporary, it plays a crucial role in improving early mobility and patient comfort within the first 24 h post-surgery.

Furthermore, we found that esketamine reduced the incidence of PONV, which was consistent with the result of the previous meta-analysis (Lin et al., 2024). It is important to note that our assessment of esketamine’s safety profile was limited. Due to inconsistent reporting across the included trials, we could not systematically analyze other clinically relevant adverse events, such as hemodynamic instability (e.g., hypertension or hypotension) or transient psychiatric symptoms (e.g., hallucinations or dysphoria). A comprehensive evaluation of the safety of perioperative esketamine, particularly in vulnerable populations like the elderly, should be a predefined and rigorously reported outcome in future large-scale trials.

The substantial heterogeneity observed (I^2^ = 53%) warrants careful consideration. This variability likely stems from clinical differences across the included trials, which are detailed in Table 1. Key sources include the type of surgery (e.g., cardiac vs. non-cardiac), varying esketamine dosing regimens (e.g., bolus doses ranging from 0.125 to 0.5 mg/kg) and timing of administration (induction vs. intraoperative infusion vs. postoperative infusion), different background anesthetic techniques, and the use of different delirium assessment tools (CAM vs. CAM-ICU vs. 3D-CAM). The limited number of studies precluded a formal meta-regression to quantify the influence of these factors, but they remain important considerations when interpreting the pooled results. We acknowledge that POD is a time-dependent outcome. Our meta-analysis synthesized the cumulative incidence of POD over each study’s entire observation period, as the reporting of time-specific incidence data (e.g., day-by-day) was inconsistent and insufficient across the included trials to permit a meaningful time-to-event or stratified analysis. This approach, while standard in many meta-analyses, may obscure variations in the timing of delirium onset.

Several important issues should be considered when interpreting our result. First and foremost, the certainty of evidence for our primary outcome, POD incidence, is low. The observed benefits must therefore be viewed as preliminary and hypothesis-generating. Second, significant heterogeneity was present, which we could not fully explain through subgroup analyses and meta-regression. Third, the TSA indicated that the required information size has not been reached, implying that the current evidence is still insufficient to draw firm conclusions.

The present meta-analysis has some limitations. First, there was considerable variability in the duration and dosage of esketamine used across the included studies, limiting the ability to conduct further subgroup analyses. Second, all studies were conducted in China; the pathophysiology of POD and the pharmacodynamic response to esketamine may be influenced by genetic, environmental, dietary, and perioperative care standards that differ across regions. This poses a profound constraint on the external validity and generalizability of our findings. Third, the positive results observed in subgroup analyses were based on a small number of studies, warranting further investigation. Therefore, larger, high-quality RCTs across diverse populations and settings are needed in the future.

## Conclusion

In conclusion, this meta-analysis suggests a potential benefit of esketamine in reducing the incidence of POD after general anesthesia, particularly in adult patients and those undergoing cardiac surgery. However, the current evidence is exclusively from China and of low certainty. Therefore, these findings should be interpreted as preliminary and hypothesis-generating ones, and definitive conclusions await confirmation in future large-scale, high-quality RCTs.

## Data Availability

The original contributions presented in the study are included in the article/Supplementary Material, further inquiries can be directed to the corresponding author.

## Supplementary material

SUPPLEMENTARY FIGURE S1 Funnel plot for postoperative delirium (POD) incidence.
