# Supplementary figures and images for: The effect of esketamine on postoperative delirium in patients undergoing general anesthesia: a systematic review and meta-analysis
Source: Front Pharmacol. 2025 Nov 7;16:1681531. doi: 10.3389/fphar.2025.1681531 (PMC12634593; doi:10.3389/fphar.2025.1681531)

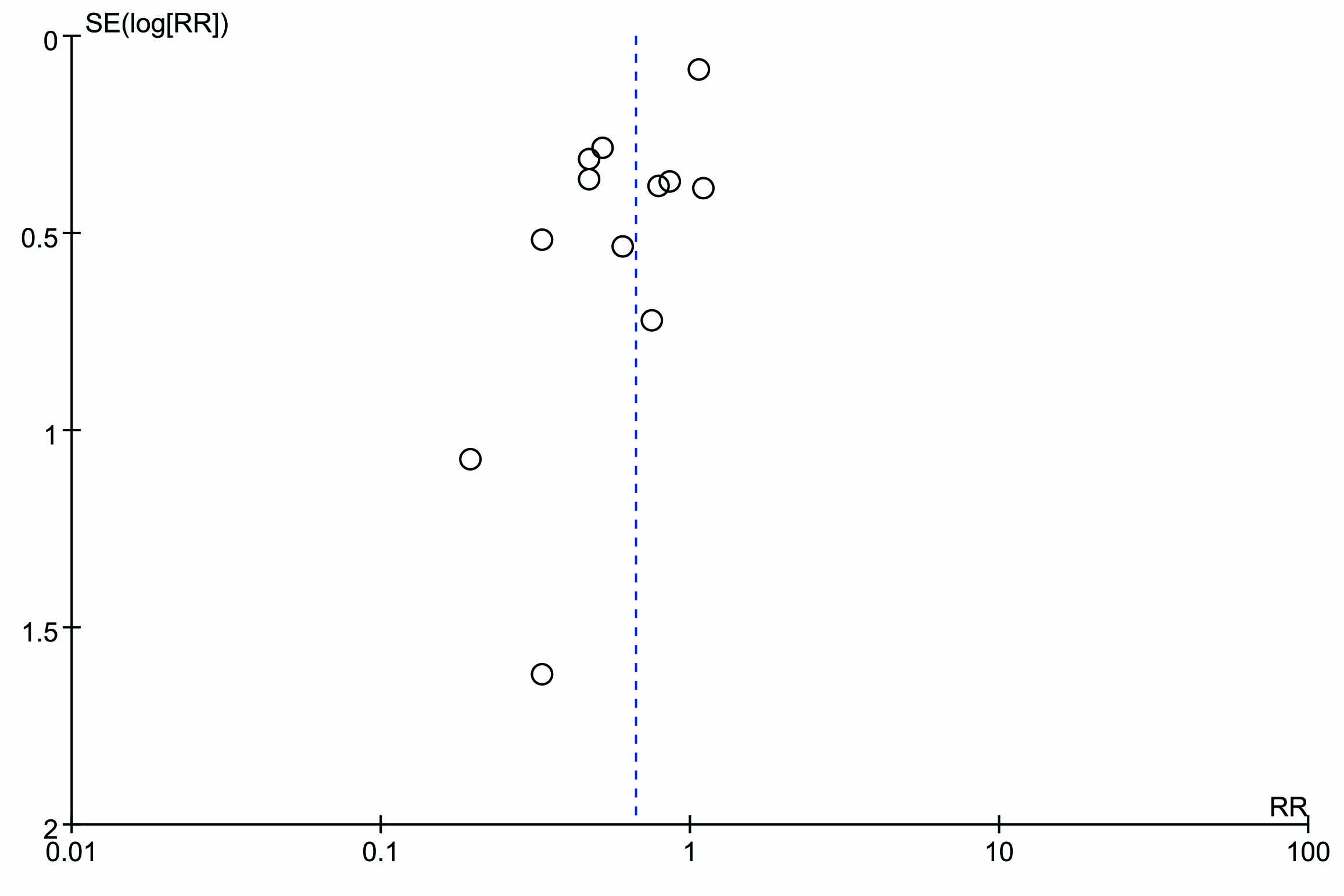

Supplement: Supplementary file 1 [file Image1.jpg]
